# Low skeletal muscle mass is predictive of dose-limiting toxicities in head and neck cancer patients undergoing low-dose weekly cisplatin chemoradiotherapy

**DOI:** 10.1371/journal.pone.0282015

**Published:** 2023-02-21

**Authors:** Jan-Niklas Becker, Robert Hermann, Jörn Wichmann, Mathias Sonnhoff, Hans Christiansen, Frank Bruns

**Affiliations:** 1 Department of Radiation Oncology, Hannover Medical School, Hannover, Germany; 2 Centre for Radiotherapy and Radiooncology Bremen and Westerstede, Westerstede, Germany; 3 Centre for Radiotherapy and Radiooncology Bremen and Westerstede, Bremen, Germany; University Hospital Zurich, SWITZERLAND

## Abstract

**Background:**

The dose-limiting effect of CT-assessed low skeletal muscle mass (LSMM) measured at the level of the third cervical vertebra has been found in head and neck cancer patients receiving high-dose cisplatin chemoradiotherapy. The aim of this study was to investigate the predictive factors for dose-limiting toxicities (DLTs) using low-dose weekly chemoradiotherapy.

**Materials and methods:**

Head and neck cancer patients receiving definite chemoradiotherapy with weekly 40 mg/m^2^ body surface area (BSA) cisplatin or paclitaxel 45 mg/m^2^ BSA and carboplatin AUC2 were consecutively included and retrospectively analysed. Skeletal muscle mass was assessed using the muscle surface at the level of the third cervical vertebra in pretherapeutic CT scans. After stratification for LSMM DLT, acute toxicities and feeding status during the treatment were examined.

**Results:**

Dose-limiting toxicity was significantly higher in patients with LSMM receiving cisplatin weekly chemoradiotherapy. For paclitaxel/carboplatin, no significance regarding DLT and LSMM could be found. Patients with LSMM had significantly more dysphagia before treatment, although feeding tube placement before treatment was equal in patients with and without LSMM.

**Conclusions:**

LSMM is a predictive factor for DLT in head and neck patients treated with low-dose weekly chemoradiotherapy with cisplatin. For paclitaxel/carboplatin, further research must be carried out.

## Introduction

A curative treatment of locally advanced head and neck cancer is radiotherapy with concurrent chemotherapy with (or without) prior surgery. Consequently, it is the only definite treatment in (technical or functional) unresectable cases. Concurrent platinum-based chemotherapy is necessary to achieve significantly higher locoregional disease control and survival [1, 2]. In particular, survival is dependent on the cumulative dose of radiation and chemotherapy [3]. Inherent treatment-associated toxicity is often a dose-limiting factor, thus negatively affecting the outcome. Identifying patients prone to a higher risk of acute toxicity is key in predicting treatment outcomes and employing adequate supportive care. This may increase the quality of life during and after treatment on the one hand and improve long-term outcomes on the other hand. Sarcopenia, defined by skeletal muscle mass (SMM) loss and function, is emerging as a predictive marker in all oncological patients for outcome and toxicities [4], especially in head and neck patients [5–7]. SMM can be determined using a single CT slide at a particular body height [8, 9]. This method has been established at the level of the third lumbar vertebra and subsequently also at the level of the third cervical vertebra to identify patients with low skeletal muscle mass (LSMM) [10, 11]. Recently, studies have shown the dose-limiting effect of LSMM measured at the level of the third cervical vertebra for primary chemoradiotherapy with high-dose cisplatin in head and neck cancer patients [12–15]. However, an increasing number of concurrent low-dose chemotherapy regimens on a weekly basis are used, achieving the same outcome with lower toxicities [16–18]. To our knowledge, no studies have evaluated the effect of LSMM on dose-limiting toxicities in head and neck cancer patients receiving low-dose weekly chemoradiotherapy. Therefore, this study investigated whether LSMM can also be used as a predictive factor for dose-limiting toxicities in low-dose weekly chemoradiotherapy of head and neck cancer patients.

## Materials and methods

### Patients and treatment

In this single-centre study, patients with head and neck squamous cell carcinoma (HNSCC) receiving definitive chemoradiotherapy with curative intent between January 2015 and October 2021 were consecutively included and retrospectively analysed. Postoperative or radiotherapy-only cases were primarily excluded. Patients for whom an accurate measurement of SMM was not possible and patients not completing treatment due to nontoxicity reasons (e.g., noncompliance or disease progression) were secondarily excluded. All patients gave their written consent. The Medical Ethical Research Committee of the Hannover Medical School approved the design of this study.

The standard treatment regimen was moderately hypofractionated volumetric arc therapy (VMAT) with a simultaneous integrated boost using a total dose of 66 Gy (daily dose, 2.2 Gy) for gross tumour volumes, 60 Gy (daily dose, 2.0 Gy) for tumour-adjacent cervical nodes at high risk for subclinical disease and 54 Gy (daily dose, 1.8 Gy) for elective cervical nodes divided into 30 fractions [19, 20]. Five fractions were applied every week for a total treatment time of six weeks. Intravenous chemotherapy was given simultaneously every week using a cisplatin target dosage of 40 mg/m^2^ body surface area (BSA), accumulating to 240 mg/m^2^ BSA for 6 weeks. Patients with contraindications to cisplatin-based chemotherapy (mainly reduced renal function) received carboplatin (AUC2) and paclitaxel (45 mg/m^2^ BSA) weekly. Only patients with those two regimens were included.

Dose-limiting toxicity was defined in cases where less than five cycles of chemotherapy were applied; thus, in the case of cisplatin, less than 200 mg/m^2^ BSA was applied during treatment. No cycle was performed in cases of haematopoietic toxicity with a white blood cell count < 3.0 x 10^9^/l and/or platelets < 75 x 10^9^/l. Cisplatin was reduced in dosage if the creatinine clearance was between 65 and 60 ml/min and discontinued below a clearance of 60 ml/min. Furthermore, in cases of infections with fever, a cycle was postponed. G-CSF was given for a white blood cell count < 1.0 x 10^9^/l. The above requirements were evaluated weekly.

### Data collection

Patient characteristics included age, sex, smoking history, p16 status in oropharyngeal tumours, tumour location, and tumour (T) and lymph node (N) stage according to the 7th edition of the American Joint Committee on Cancer (AJCC) Staging Manual [21]. In addition, every week before any planned chemotherapy administration, weight and height were measured along with blood samples, including serum creatinine concentration (mg/dl) and a complete blood count. Furthermore, feeding status and toxicities were noted according to the Common Terminology Criteria for Adverse Events (CTCAE) v5.0 [22] before and during the treatment on a weekly basis in the individual patient protocol.

### CT-based sarcopenia analysis

SMM was assessed by CT scans (3 mm slices) for radiation planning performed on a SOMATOM Definition AS CT (Siemens, Germany) directly before treatment. A thermoplastic mask was used to ensure immobilization. Following the validated procedure of Swartz et al. [11, 23], the first slide, including the entire vertebral arc scrolling from cranial to caudal at the level of the third cervical vertebra, was selected and manually followed by delineation of the cross-sectional area (CSA) of both sternocleidomastoid and paravertebral muscles using the software Centricity PACS Radiology RA1000 Workstation (GE Healthcare, Barrington IL, USA), see “S1 Fig”. Patients with scans with severe dental artefacts or tumour infiltration of the relevant muscles at the level of the third cervical vertebra that impeded accurate assessment of CSA were excluded. Next, the CSA of both sternocleidomastoid and paravertebral muscles was added to obtain the respective skeletal muscle CSA on the third cervical vertebra. This was further used to calculate the estimated skeletal muscle CSA on the third lumbar vertebra according to the formula by Swartz et al. [11]. The CSA (cm^2^) was divided by the square of the body height (m^2^) to adjust for patients’ stature. This results in the skeletal muscle index (SMI, cm^2^/ m^2^) at the third lumbar vertebra which is considered a comparable surrogate marker for the SMM [24]. The continuous SMI was additionally utilized to categorize patients for LSMM corresponding to a previously established cut-off value of SMI < 43.2 cm^2^/m^2^ [15, 25]. In further analysis, LSMM is therefore defined as SMI < 43.2 cm^2^/m^2^. Delineation was performed by an experienced single researcher (JNB) [26]. Imaging analysis was further tested in terms of the reproducibility of the study by measuring inter- and intraobserver reliability [27]. Interobserver reliability was determined by comparing the SMI measured by two observers (JNB and JW) from 25 randomly selected patients. Intraobserver reliability was determined by comparing two sets of measurements of 25 randomly reselected patients performed by a single observer separated by two weeks. The observers were blinded to previous delineations.

### Statistical analysis

Patients were stratified by LSMM status and described with descriptive statistics. Data are presented as the mean with standard deviation for normally distributed continuous variables and as a number with percentage for categorical variables. Normality was confirmed using the Kolmogorov–Smirnov test and Q-Q-plots. The Pearson chi-square test was used for categorical group comparisons, and the independent sample t test was used for continuous variables. Univariate and multivariate logistic regression analyses were used to assess the predictive value of LSMM and DLT. Variables with a p value lower than 0.05 (two-tailed) in univariate analysis were selected for inclusion in multivariate analysis. The goodness of fit of the multivariate model was analysed using the Hosmer–Lemeshow test. The imaging analysis of the relevant CSA was tested for reproducibility by measuring inter- and intraobserver reliability with respective interclass correlation coefficients (ICCs). An ICC > 0.9 was considered excellent according to Koo et al. [28]. Statistical significance was considered at p < 0.05. There were no missing data. All statistical analyses were conducted with IBM Statistical Package for Social Sciences version 27.

## Results

In total, 109 patients were found to be receiving primary chemoradiotherapy. Four patients did not complete treatment for nontoxic reasons, and eight patients had inadequate image quality and were excluded. Analysis was performed on the remaining 97 patients.

### Patient characteristics

The characteristics of the low-skeletal muscle patients are presented in Table 1. All continuous variables were normally distributed.

**Table 1:** Characteristics of included patients (n = 97).

	Without low SMM (*n* = 55)	Low SMM (*n* = 42)	*P*-value
Sex			**<0.01** ^a^
Female	0 (0%)	19 (45.2%)	
Male	55 (100%)	23 (54.8%)	
Age at diagnosis (years)	61.1 (±8.5)	64.4 (±8.2)	0.06 ^b^
Smoking			
No	24 (43.6%)	17 (40.5%)	0.76 ^a^
Yes	31 (56.4%)	25 (59.5%)	
Weight (Kg)	83.3 (±18.4)	62.1 (±12.6)	**0.01** ^b^
Length (cm)	175.3 (±6.4)	172.3 (±8.5)	**0.03** ^b^
SMI (cm^2^/m^2^)	50.2 (±6.5)	37.1(±5.5)	<**0.01** ^b^
P16 status (oropharyngeal tumour)			0.24 ^a^
Positive	7 (38.9%)	3 (20%)	
Negative	11 (61.1%)	12 (80%)	
Tumour site			0.06 ^a^
Oropharynx	18 (32.7%)	15 (35.7%)	
Larynx	6 (10.9%)	2 (4.8%)	
Hypopharynx	14 (25.4%)	5 (11.9%)	
Oral cavity	12 (21.8%)	19 (45.2%)	
Nasopharynx	5(9.1%)	1 (2.4%)	
T-stage*			0.61 ^a^
T1	2 (3.6%)	2 (4.8%)	
T2	11 (20%)	5 (11.9%)	
T3	17 (30.9%)	11 (26.2%)	
T4	25 (45.5%)	24 (57.1%)	
N-Stage*			0.40 ^a^
N0	13 (17.5%)	7 (16.7%)	
N+	42 (82.5%)	35 (83.3%)	
Anatomic stage group*			0.76 ^a^
Stage II	8 (14.5%)	4 (9.5%)	
Stage III	11 (20%)	9 (21.4%)	
Stage IV	36 (65.5%)	29 (69.1%)	

Categorical data with the percentage of total group size *n*. Continued data (age, weight, and length) is given as the mean with standard deviation. The T stage is categorizing the tumour size while the N stage represents the presence of lymph node metastasis (N+ = at least one positive lymph node).

Significance *p* was calculated by ^a^ Pearson’s chi-squared test and ^b^ Student independent t test.

*Staging confirm with the AJCC Cancer Staging Manual, 7th edition [21].

All female patients were classified into the low skeletal muscle mass group (LSMM). Mean age was slightly higher and mean weight significantly lower in the LSMM group. Mean height was also significantly lower in the LSMM group. The primary tumour sites were the oropharynx (32.7%) in the normal SMM group and the oral cavity (45,2%) in the LSMM group. T and N as well as anatomic staging were not significantly different between normal and low SMM.

### Dose limitations and toxicities

Dose limitations were observed significantly more often in the LSMM group (35.7%) than in the normal SMM group (10.9%) presented in Table 2. Significant differences were documented in the cisplatin subgroup. However, in the paclitaxel/carboplatin subgroup, no significant differences were observed between the groups. The reason for dose limitation was always haematopoietic toxicity in patients without LSMM. In low SMM patients, two-thirds also had dose limitations due to haematopoietic toxicity, nephrotoxicity (n = 2), and infections (n = 3). In five patients a cisplatin dose reduction was necessary due to reduced renal function during treatment (three patients with one reduced cycle, two patients with two reduced cycles). Those patients had also less than four cycles of cisplatin each, thus, dose-limiting toxicity anyway.

**Table 2:** Dose-limiting toxicity (DLT) according to SMM status.

Total (*n* = 97)	Without low SMM (*n* = 55)	Low SMM (*n* = 42)	*P*-value
DLT			**<0.01**
No	49 (89.1%)	27 (64.3%)	
Yes	6 (10.9%)	15 (35.7%)	
DLT in cisplatin			
No	38 (90.5%)	20 (66.7%)	**0.01**
Yes	4 (9.5%)	10 (33.3%)	
DLT in paclitaxel/carboplatin			0.14
No	11 (84.6%)	7 (58.3%)	
Yes	2 (15.4%)	5 (41.7%)	
Reason of DLT			0.45
Haematopoietic toxicity	6 (100%)	10 (66.7%)	
Nephrotoxicity	0	2 (13.3%)	
Infection	0	3 (20%)	

DLT dose limiting toxicity; SMM skeletal muscle mass; Significance *p* calculated by Pearson’s chi-squared test

The number of patients with a feeding tube prior to treatment was not significantly different in patients with and without LSMM as shown in Table 3. However, dysphagia before treatment was significantly higher in LSMM patients. No significant difference between normal and low SMM was found in relation to weight loss during treatment or mucositis, dermatitis, and dysphagia at the end of the treatment.

**Table 3:** Toxicities and feeding status according to SMM status.

	Without low SMM (*n* = 55)	Low SMM (*n* = 42)	*P*-value
Mucositis*			0.41
I/II	49 (89.1%)	35 (83.3%)	
III/IV	6 (10.9%)	7 (16.7%)	
Dermatitis*			0.98
I/II	51 (92.7%)	39 (92.9%)	
III/IV	4 (7.3%)	3 (7.1%)	
Dysphagia*			0.11
I/II	23 (41.8%)	11 (26.2%)	
III/IV	32 (58.2%)	31 (73.8%)	
Dysphagia before treatment			**0.01**
I/II	48 (87.3%)	27 (64.3%)	
III/IV	7 (12.7%)	15 (35.7%)	
Weight loss during treatment			0.40
<10%	45 (81.8%)	37 (88.1%)	
> = 10%	10 (18.2%)	5 (11.9%)	
Feeding-tube prior treatment			0.46
No	38(69.1%)	26(61.9%)	
Yes	17(30.9%)	16(38.1%)	

SMM skeletal muscle mass; Significance *p* calculated by Pearson’s chi-squared test, Mucositis, Dermatitis, and Dysphagia grouped according to CTCAE v5.0 [22]

*highest CTCAE classification during treatment

Table 4 presents the results of the univariate and multivariate logistic regression for DLT. Significant for predicting DLT were older age (1.08 95% CI 1.01–1.15) and LSMM (4.54 95% CI 1.58–13.06). In multivariate analysis, only LSMM was a significant predicting factor for dose-limiting toxicity, giving an odds ratio of 3.96 (95% CI 1.34–11.64). The Hosmer–Lemeshow test gave a high goodness-of-fit with a Chi-Square = 8.615 and a p = 0.376.

**Table 4:** Univariate and multivariate logistic regression for DLT.

	Univariate analysis Odds ratio (95% CI)	*P*-value	Multivariate analysis Odds ratio (95% CI)		*P*-value
Gender					
Female	Ref				
Male	1.05 (0.31–3.57)	0.90			
Age	1.08 (1.01–1.15)	**0.02**	1.07 (1.00–1.14)	0.06	
BMI	1.01 (0.93–1.10)	0.86			
Anatomic stage group					
Stage II	Ref				
Stage III	0.73 (0.14–3.72)	0.70			
Stage IV	1.21 (0.38–3.92)	0.75			
Dysphagia before treatment					
I/II	Ref				
III/IV	1.32 (0.39–4.43)	0.65			
LSMM					
No	Ref				
Yes	4.54 (1.58–13.06)	**<0.01**	3.96 (1.34–11.64)	**0.01**	

DLT dose limiting toxicity; LSMM low skeletal muscle mass; BMI body mass index; CI confidence interval; Significance *p*

### Inter- and intraobserver reliability

The calculated ICC was 0.97 (95% CI 0.92–0.99) for interobserver reliability and 0.98 (95% CI 0.95–0.99) for interobserver reliability. Both are excellent, with values above 0.9.

### Discussion

Our study identified LSMM as a predictor for dose-limiting toxicity in weekly low-dose concurrent cisplatin chemoradiotherapy for HNSCC patients. These results are in line with previous studies using high-dose cisplatin chemotherapy regimens.

Bril et al. [14] found a trifold risk of DLT due to LSMM in HNSCC patients undergoing high-dose chemoradiotherapy. They proposed that a low-dose weekly regimen may be better in sarcopenic patients. In our study, the risk of dose-limiting toxicity was even slightly higher in LSMM patients. Our results, therefore, argue against a benefit of a low-dose weekly regimen in the LSMM group.

In our study, patients who were not eligible for weekly chemotherapy with 40 mg/m^2^ cisplatin received carboplatin AUC2 and paclitaxel 45 mg/m^2^ BSA as an alternative. Here, LSMM could not be identified as a statistically relevant predictive factor for dose-limiting toxicity. However, the group size of carboplatin/paclitaxel patients was relatively small compared to that of the cisplatin group. On the other hand, the respective percentage differences tended to be lower in the carboplatin/paclitaxel group. This finding may have clinical relevance in choosing a therapy regimen for LSMM patients. Better tolerance may result in a better outcome. A possible explanation could be the carboplatin dosage according to the AUC2 instead of the body surface area used for cisplatin [29]. Another possible explanation is that hydrophilic cisplatin is distributed mainly in muscle rather than fat. Therefore, patients with LSMM but normal or high body mass due to fat may receive a relative overdosage of BSA-calculated cisplatin, resulting in more toxicity [30]. However, regarding the LSMM paclitaxel/carboplatin group percentage, relatively more patients had a DLT than in the cisplatin group. This finding could be due to more vulnerable patients in the paclitaxel/carboplatin group. No published research using weekly chemotherapy with the paclitaxel/carboplatin regimen alone or with paclitaxel/carboplatin stratification was available regarding DLT in HNSCC patients. Therefore, we focused on the cisplatin group regarding dose limitation, for whom data are available.

Comparing the relative amounts of patients with cisplatin dose limitation, thus less than 200 mg/m^2^ BSA for the total treatment, our results align with previous studies using the high-dose cisplatin regimen. Cargi et al. [13] had nearly the same ratios for patients with and without LSMM. Additionally, in a meta-analysis comparing weekly low-dose and high-dose cisplatin regimens, no significant differences were found between both regimens in reaching the cumulative 200 mg/m^2^ BSA dosage in the definite chemoradiotherapy of HNSCC patients [18]. Dosage below this landmark has been shown to give inferior results [1, 2]. Reaching 200 mg/m^2^ BSA with two cycles of a high-dose or five cycles of a weekly low-dose cisplatin regimen seems to have no impact on progression-free and overall survival. Nevertheless, the different regimens have been shown to impact treatment toxicities.

In definitive and postoperative chemoradiotherapies of HNSCC, a weekly cisplatin regimen has been shown to cause less severe toxicities [18, 31]. In the study of Cargi et al. [13], using a high-dose cisplatin regimen, mostly ototoxicity and nephrotoxicity were reasons for a dose limitation. In our study, in contrast, mainly haematopoietic toxicity led to a reduction in the number of dose cycles. This is consistent with previous studies [18]: in the high-dose cisplatin regimen, acute severe nephrotoxicity (Grade 3 and 4 according to the CTCAE [22]) was significantly more frequent compared to the weekly regimen. Data were also found only in studies with high-dose cisplatin for severe ototoxicity. Therefore, a clear argument for using the weekly regimen during chemoradiotherapy is causing less severe toxicities with no inferiority in terms of progression-free survival and overall survival. Here, one must also consider the toxicities more related to radiation.

Consistent with previous publications [32, 33], in our study, LSMM could not be identified as a predicting factor for skin and mucosa toxicities or dysphagia at the end of treatment. These typical skin and mucosa responses are mostly due to local radiation. Based on our current knowledge, these reactions can only be reduced by a well-balanced dose plan for these vulnerable regions and intensified supportive care during treatment [34, 35]. Regarding the initial feeding status, significantly more patients with LSMM already had severe dysphagia before treatment. This result is also in line with previous studies [12] and, incidentally, even not surprising, as malnutrition is a major reason for sarcopenia [36].

Interestingly, feeding tube placements before treatment were not significantly higher in LSMM patients in our study population. However, weight loss was not significantly different between LSMM and non-LSMM patients during chemoradiotherapy. It is possible that more non-LSMM patients received a prophylactic feeding tube without severe dysphagia. This aspect may need further investigation. Indeed, dedicated nutrition combined with physiotherapy helps LSMM patients gain more crucial muscle mass before and perhaps during treatment [37]. This could result in fewer dose-limiting toxicities and consequently a better outcome. However, the placement of feeding tubes with a relatively high risk of complications could be evaluated more critically using the LSMM [38, 39].

A strength of our study compared to all studies published before was the inclusion of only definitive chemoradiotherapy treatment. In previous studies, differences between postoperatively and definitely treated patients were, as far as they were analysed, always significant in terms of low skeletal muscle mass [13]. Thus, the results derived from a group including both could be less reliable for each part of the group and under- or overestimate the dose-limiting effect of LSMM, for instance. In previous studies, different concurrent chemotherapy or even immunotherapy regimens were often not stratified. In this study, we were the first to distinguish between chemotherapeutic agents, making the results more comparable. Intra- and interobserver reliability was confirmed to make the measurements more robust.

As with most previous studies, the retrospective nature of this study has some intrinsic limitations. Hearing function was measured mostly before treatment but not during or after treatment. Even though no patients stated an evident subjective hearing loss during the treatment in our records, regular measurements could have been compared with other studies in terms of ototoxicity. Patient assessment and blood samples were systematically collected weekly during treatment. Data in the weeks after therapy in terms of toxicities were not available. This is also true in previous studies, but LSMM may influence the length of specific toxicities and thus the recovery from such therapy [12].

Furthermore, the relatively small number of patients receiving paclitaxel and carboplatin instead of cisplatin only can give a trend but is too small for a clear statement based on a statistical calculation. The cut-off for LSMM was used from published studies for better comparison. Using this cut-off, all women fell into the LSMM group. The debate about suitable cut-off values is still ongoing [40].

## Conclusions

This is the first study showing that low skeletal muscle mass by CT-scan measurements of the third cervical vertebra is a predictor for dose-limiting toxicity in concurrent chemoradiotherapy using weekly low-dose cisplatin-based regimens in HNSCC patients. Further research should be carried out on different chemotherapy agents and chronic toxicities.

## Supporting information

S1 Fig Skeletal muscle measurements. Axial neck CT at the level of the third cervical vertebra with skeletal muscle measurements. (1) cross-sectional area (CSA) of the right sternocleidomastoid muscle, (2) CSA of the left sternocleidomastoid muscle, and (3) CSA of the paravertebral muscles. **a** Patient with low skeletal muscle mass. **b** Patient without low skeletal muscle mass.
(DOCX)
