# Supplementary material for: Low skeletal muscle mass is predictive of dose-limiting toxicities in head and neck cancer patients undergoing low-dose weekly cisplatin chemoradiotherapy
Source: PLoS One. 2023 Feb 21;18(2):e0282015. doi: 10.1371/journal.pone.0282015 (PMC9942991; doi:10.1371/journal.pone.0282015)
Supplement: S1 Fig — Axial neck CT at the level of the third cervical vertebra with skeletal muscle measurements. (1) cross-sectional area (CSA) of the right sternocleidomastoid muscle, (2) CSA of the left sternocleidomastoid muscle, and (3) CSA of the paravertebral muscles. a Patient with low skeletal muscle mass. b Patient without low skeletal muscle mass. (DOCX) [file pone.0282015.s001.docx]

**S1 Fig. Skeletal muscle measurements**


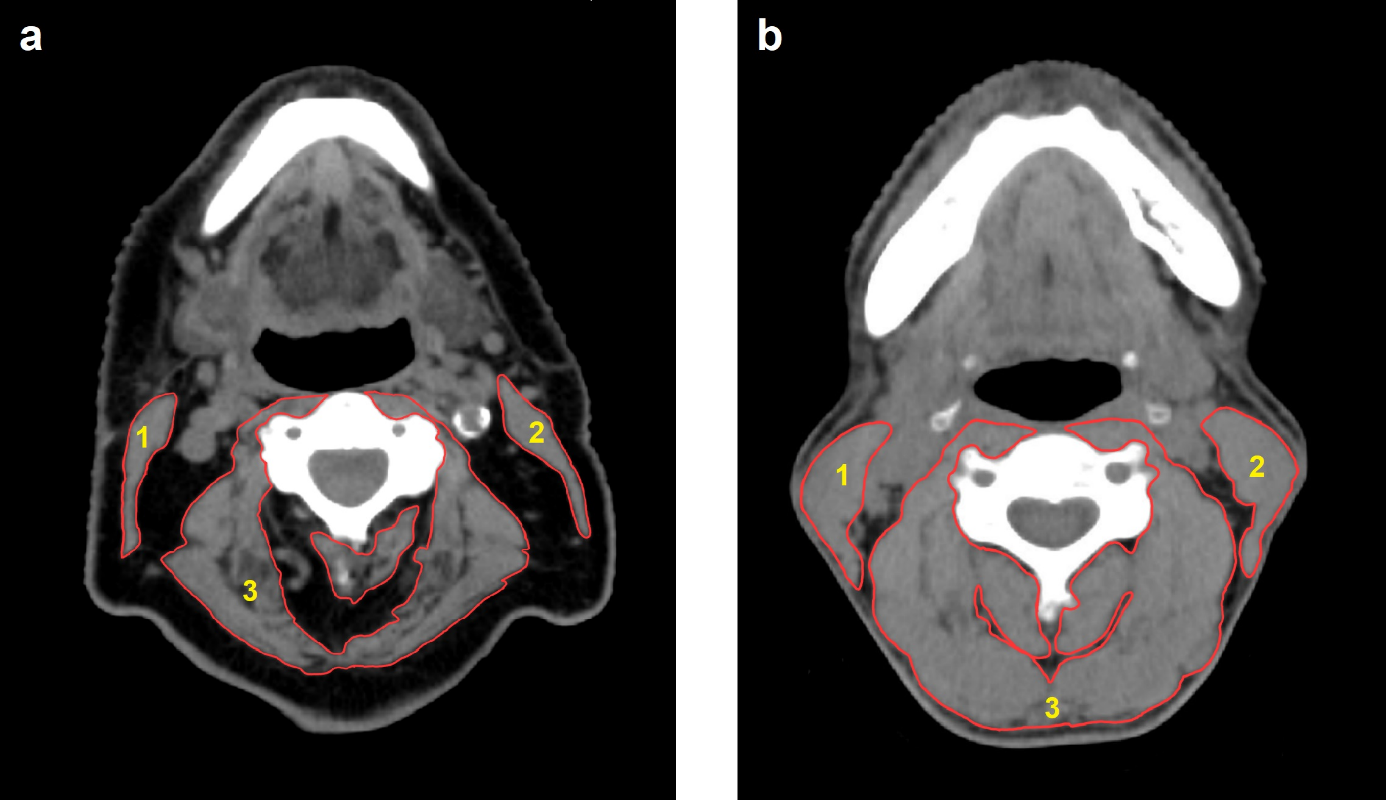
 Axial neck CT at the level of the third cervical vertebra with skeletal muscle measurements. (1) cross-sectional area (CSA) of the right sternocleidomastoid muscle, (2) CSA of the left sternocleidomastoid muscle, and (3) CSA of the paravertebral muscles. **a** Patient with low skeletal muscle mass. **b** Patient without low skeletal muscle mass.
